# From mobile crowdsourcing to crowd-trusted food price in Nigeria: statistical pre-processing and post-sampling

**DOI:** 10.1038/s41597-023-02211-1

**Published:** 2023-07-12

**Authors:** Giuseppe Arbia, Gloria Solano-Hermosilla, Vincenzo Nardelli, Fabio Micale, Giampiero Genovese, Ilaria Lucrezia Amerise, Julius Adewopo

**Affiliations:** 1 Catholic University of Sacred Heart, Rome, Italy; 2 University Pablo de Olavide, Sevilla, Spain; 3 University of Milan-Bicocca, Milan, Italy; 4 European Commission, Joint Research Centre, Seville, Spain; 5 European Commission, Joint Research Centre, Ispra, Italy; 6 University of Calabria, Rende, Italy; 7 International Institute for Tropical Agriculture (IITA), Lagos, Nigeria

**Keywords:** Agriculture, Developing world

## Abstract

Timely and reliable monitoring of food market prices at high spatial and temporal resolution is essential to understanding market and food security developments and supporting timely policy and decision-making. Mostly, decisions rely on price expectations, which are updated with new information releases. Therefore, increasing the availability and timeliness of price information has become a national and international priority. We present two new datasets in which mobile app-based crowdsourced daily price observations, voluntarily submitted by self-selected participants, are validated in *real-time* within spatio-temporal markets (pre-processed data). Then, they are reweighted weekly using their geo-location to resemble a formal sample design and allow for more reliable statistical inference (post-sampled data). Using real-time data collected in Nigeria, we assess the accuracy and propose that our reweighted estimates are more accurate with respect to the unweighted version. Results have important implications for governments, food chain actors, researchers and other organisations.

## Background & Summary

Timely and reliable monitoring of food market prices at high spatial and temporal resolution is essential to understanding market and food security developments and supporting timely policy and decision-making. Yet, in a time of new digital technologies and “big data” approaches, one significant challenge in producing statistics is the trade-off between the timeliness of new alternative data sources and the accuracy of traditional sample survey data^1,2^. The reference here is to the concept advanced by^3^, which classifies “big data” types as (i) data generated by people and stored in a digitalised format (e.g. from mobile apps, Twitter), (ii) data produced automatically by people when interacting with IT systems (e.g. scanner data) and (iii) machine-generated data usually captured by sensors. Official, reliable price estimates are often released several weeks after the end of the month or the quarter, and usually, as indices (e.g. the Consumer Price Index) aggregated at the regional or national level. Governments, agencies and market participants make decisions based on price expectations that are updated as information becomes available, which needs to be up-to-date and transparent—quality and trusted data that can be accessed in time by all market players and stakeholders^4^— to increase efficiency and market integration^5^ and reduce uncertainty around decision-making^6^. While market efficiency (or vertical market integration) refers to the transmission of price signals from one marketing channel to another, in spatially well-integrated markets, price signals (informing producers and consumers) are transmitted from supply-deficit regions to surplus markets. For this reason, increasing availability and timeliness of market price information has become a priority at the international level, in both developed^7^ and developing economies^8^, and at national levels—see, e.g. review of food prices observatories and the European Food Prices Monitoring Tool^9^.

In the statistical tradition, following the seminal contributions of Neyman^10^ and Fisher^11^ and the well-documented failure of non-probabilistic samples^12^, surveys commonly involve collecting probability samples, which can guarantee sound probabilistic inference^13^. However, new Information Communication Technologies (ICTs) and innovative data sourcing methodologies (such as web scraping, scanner data, online surveys, mobile apps/mobile phone crowdsourcing, Internet-Of-Things, smart meters, internet panels and other citizen participatory approaches) offer the potential to complement official statistics with much higher data frequency and higher spatial granularity^14,15^. As such, *crowdsourcing*, in the form of data voluntarily collected by individuals using a mobile app, is becoming very popular in the empirical literature.

In particular, in Africa, the rapid development and spreading of mobile phone networks, mobile internet and increasing smartphone penetration have given rise to several projects aiming to collect market food prices using mobile phones and citizen participation^16–20^.

However, data quality is one of the most critical data management issues as data from various sources and formats become available to ensure the usability of the data^21^. A characteristic shared by these new emerging types of data sources is represented by the uncontrolled presence of both sampling and non-sampling errors.

The first problem concerns the lack of any precise statistical sample design. In particular, with crowdsourcing data collection, participation is voluntary, thus producing self-selection of the data collectors. This situation is described in statistics as “convenience sampling” (a type of non-probabilistic sampling approach), which famously does not permit statistical inference^13^. More precisely, in a formal sample design, the choice of observations is suggested by a precise mechanism, which allows the probabilities of inclusion of each unit to be calculated (and, hence, sound inferential results). On the contrary, with *convenience* sampling, no probability of inclusion can be calculated, thus giving rise to over- or under-representativeness of the sample units^22,23^. Moreover, non-sampling errors such as measurement errors and imprecisions are also frequent in these non-traditional types of data sources. But comparatively less attention has been paid to developing new quality approaches to produce trustable datasets from these emerging sources (for exceptions, see e.g.^24–27^).

As a matter of fact, before considering this data as a new source of trustworthy statistics, it is necessary to address both sampling and non-sampling errors adequately. This paper aims to present a pre-processing and aggregation approach to correcting, in near (referring to the delay introduced by automated data processing between raw data submission and dissemination of processed data) real-time, the crowdsourcing data sample estimates, taking both problems into account. In particular, when dealing with sampling errors, we will introduce a reweighting procedure to minimise the bias and inefficiencies connected with convenience sampling. Reweighting procedures are quite common in surveys, even when they rely on probabilistic samples. Standard approaches include post-stratification, generalised regression estimation (GREG) and calibration. Post-stratification is a common strategy in which we assign different weights to each sample unit after the fact so that the weighted sample matches some population characteristics^28,29^. GREG is an extension of post-stratification used to ensure that the weighted sum of each variable corresponds to the total population value^30^. Similarly, calibration involves adjusting weights from the probabilistic design to match the known population totals^31–33^. have shown how calibration can also be used for non-probabilistic samples. In a probabilistic design setting, all these methods are used to increase efficiency and reduce bias by adjusting for the under- or over-representation of specific sub-groups that constitute the sample.

We are working in the same tradition to suggest a reweighting procedure based on the calculation of weights obtained through a comparison between the data available from voluntary data collection and a desired sample drawn according to some probabilistic procedure. We call this procedure **post-sampling**^34^. Our work is empirically motivated firstly by the innovative initiatives launched to collect real-time food price data, particularly in developing countries, following the agricultural and food price hikes and volatility of 2007–2008 and 2011. More recently, the COVID-19 pandemic in 2020 has underlined the need for real-time food price information for rapid and targeted food security interventions. Secondly, it is motivated by researchers’ and practitioners’ recognition of the consequent need to develop new effective quality assurance methods for new data sources^20^. We apply the methodology to the set of prices submitted through a mobile app in Nigeria by citizen volunteers between April 2021 and November 2021, developed by the Food Price Crowdsourcing Africa (FPCA) platform launched by the European Commission in 2018. An example of its application in price analysis during the pandemic can be found in^35^. The contribution of this paper is twofold. First, it introduces and applies a method to deal in real-time with sampling and non-sampling errors in price crowdsourcing. Second, it provides a food price dataset of high frequency and spatial granularity in Nigeria that demonstrates the potential of crowdsourcing to complement conventional data sources and opens the door to new studies on the spatial dynamics of prices. The paper is laid out as follows.

The section on Methods describes the data, discusses solutions to the problem of reducing non-sampling errors by identifying standard and spatial outliers, and presents the post-sampling strategy. The following section presents the data records associated with this work, including the repository where this information is stored. The next section discusses the technical validation through an empirical application, and the final section contains information on the availability of the code.

## Methods

### Collecting and pre-processing crowdsourced food prices

This section presents the dataset we will use for the case study discussed in the technical validation section. Notably, our work refers to a dataset collected by the Food Price Crowdsourcing Africa (FPCA) platform, launched in 2018 by the European Commission’s Joint Research Centre (EC-JRC), the crowdsourcer or requester, to test “on the ground” innovative crowdsourcing-based (relying on voluntary citizen contributions, the crowd) data-gathering systems and statistical approaches to collect and disseminate reliable and geolocated real-time food prices in a cost-effective way. Developed with the International Institute for Tropical Agriculture (IITA) in Nigeria and Wageningen UR, the tool includes an open-source mobile app linked to a data platform, a tested incentive system—monetary and non-monetary (e.g. “nudges”)^36^— and an algorithm to automatically process and validate citizen data on food prices at different points in the value chain (i.e. farm gate, wholesale and retail), covering both urban and rural areas^34^. The data is published in near real-time on a web dashboard^37^. The tool initially covered four commodities (rice, maise, beans and soybeans) and their varieties and two states in north-west Nigeria (Kano and Katsina), and in 2021 (FPCA’s second wave or FPCA-II) expanded to an additional commodity (garri) and two states (Kaduna and Lagos). The tool provides two types of datasets. First, data covering individuals’ demographic characteristics, such as age, gender, occupation, and household size, is taken from the registration form in the mobile app. This data also contained information on how the collector learnt about the initiative, their motivation for participating and their preferred way of exchanging information. These auxiliary variables can be crucial to analyse data quality in relation to crowd characteristics or how to motivate participation better. Second, data on market transactions (price submissions), such as geo-coordinates, commodity, quality grade, price—expressed in the local currency, the Naira (₦), equivalent to 0.002867 Euro during the implementation of FPCA^38^—, packaging volume, market type and distance to market, is taken from the mobile app submission form. In exchange for a gamified monetary reward, volunteers were asked to submit actual transaction prices (paid/obtained), although the mobile app also allowed them to submit prices as mere observers. In this study, we used the second type of data: the actual data submissions between April 2021 and November 2021 in the states of Kano, Katsina, Kaduna and Lagos and used Local Governmental Area (LGA) and state aggregation levels. Notably, the LGAs correspond to the Second Administrative Level Boundaries, developed by the United Nations^39^ to promote the availability of reliable geospatial information for sustainable development (i.e. policy-making, programming, and operations) and knowledge- and information-sharing and the state corresponds to the First Administrative Level Boundaries.

**Fig. 1:**
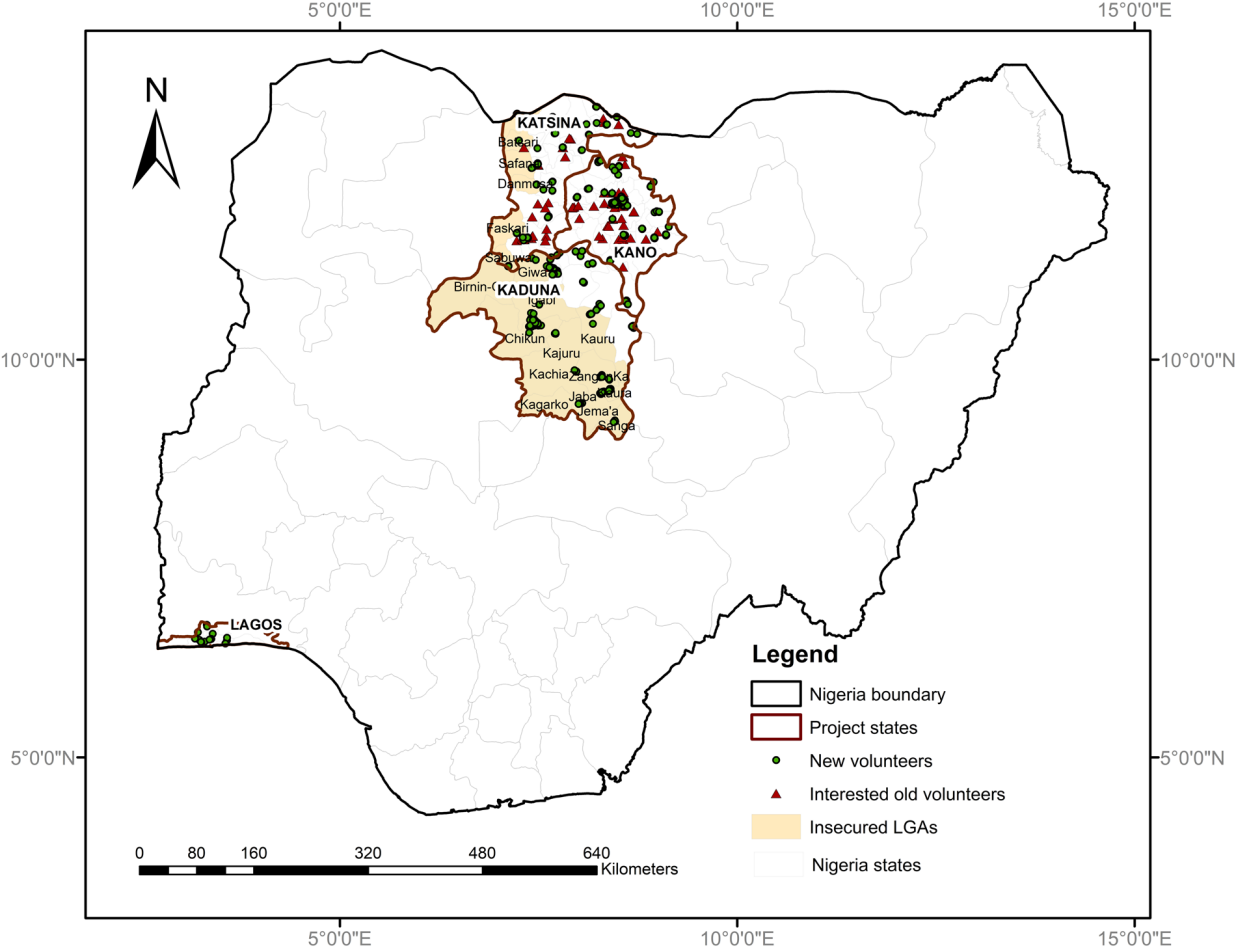
Map showing the focal states of the FPCA project and the spatial distribution of the volunteers within the focal states during the project’s second phase (FPCA-II) from April to November 2021.

A total of 904 volunteers from a crowd of 1306 registered and several unregistered volunteers submitted more than 26,700 data records (each may contain several prices for several food products) consisting of 230,335 daily market price observations during their routine market visits. The result was a weekly average of 6,398 price observations, with a remarkably declining trend over time, showing that attracting the crowd is easier than retaining it^40–42^ and nine food product varieties per data submission. Furthermore, they submitted data whenever they wanted to, constituting a convenience and, thus, a *non-probabilistic sample*. The crowdsourcing exercise aimed to assess the potential of this form of data collection and to establish a quality methodology to efficiently produce reliable geo-referenced data on food prices at the local and regional level, accessible in near real-time, in order to meet the data needs of governments, food supply chain actors and other institutions. Figure 1 shows the spatial distribution of the crowd volunteers in the focal states.

**Fig. 2:**
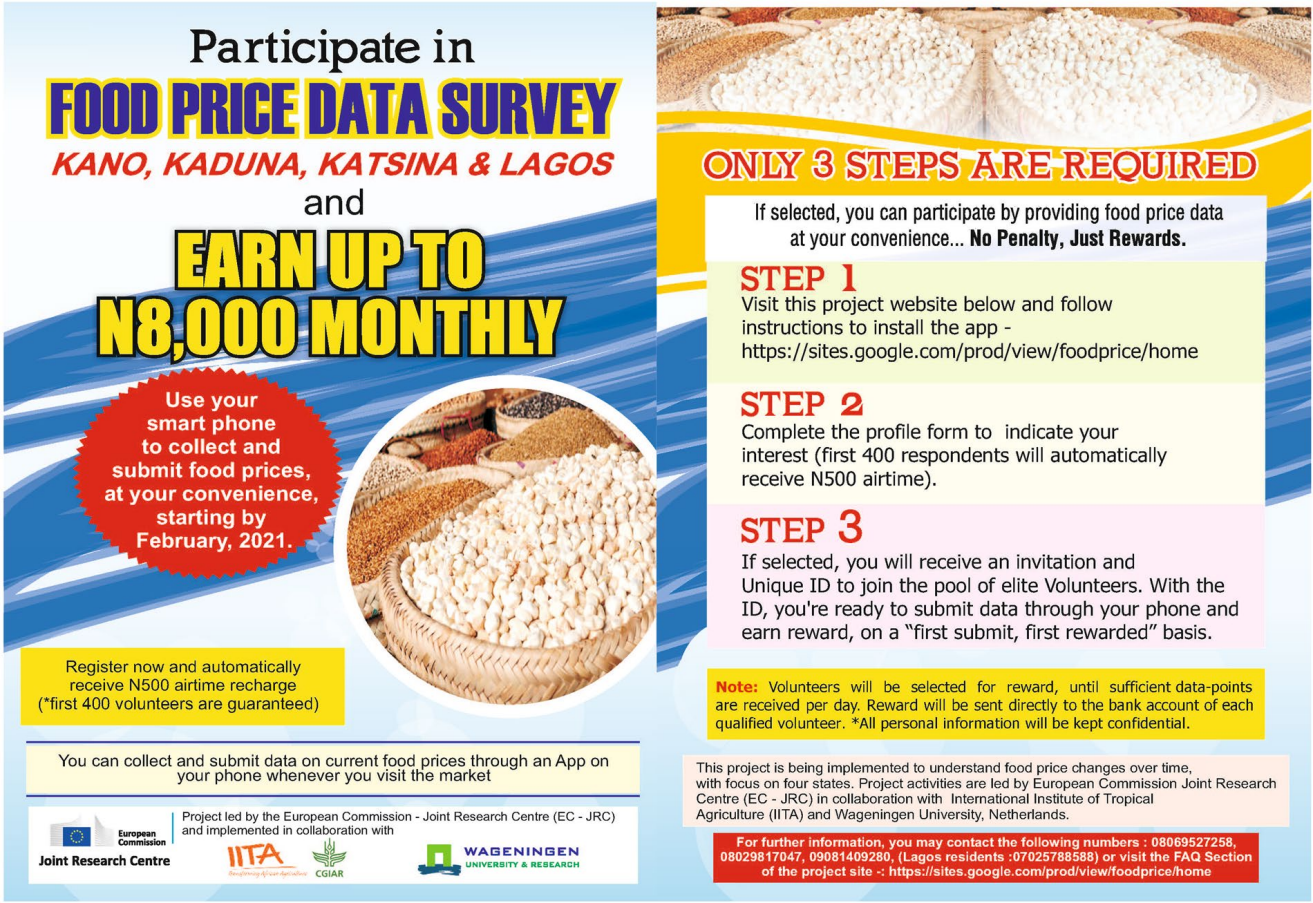
Flyer distributed to invite prospective volunteers to participate in the second wave of food price crowdsourcing in Africa (FPCA-II) project in Nigeria.

To achieve adequate population coverage, the FPCA made use of the fact that accessible broadband mobile technology was available in most parts of the selected states, and an increasing number of people were using smartphones both within and outside cities. As such, the mobile phone penetration rate can be used as a proxy for the coverage of the target population of individuals^3^. This offered an excellent opportunity for crowdsourcing using mobile phones. Radio adverts (only in FPCA-I) and flyers served to advertise the initiative (Fig. 2). Additionally, the collaboration of governmental agricultural extension agents, social media, and word of mouth contributed to increasing awareness. Crowd volunteers were only required to own a smartphone with GPS and follow online instructions. The system was based on Open Data Kit (ODK) and deployed on a compatible cloud-based server, ONA, which stores data submitted through the mobile app that is available via a private Application Programme Interface (API) in real-time.

To increase the willingness of the crowd to participate, the initiative included a gamified reward system where *valid* daily submissions were rewarded (4 € per submission) on a “first-submit, first-rewarded” basis up to the 30^th^ submission. So it encouraged immediate (real-time) submission, with weekly and monthly limits to reduce potential fraud (e.g. sending repeated numbers). The initiative also included behavioural tools such as “nudges” (i.e. information sent to the crowd that may influence their behaviour without restricting their freedom of choice^43^) via SMS messages. For example, the SMS shared “social norms” (the number of prices submitted by peer volunteers) and disclosed aggregated price information from the crowdsourced dataset by sharing the link to the web dashboard^36^.

From a statistical perspective, mobile phone numbers are not an ideal sample frame for observing units from a target population as different links can be established between mobile phone numbers and individuals (e.g. one-to-one, one-to-many, many-to-one^3^). To establish a one-to-one relationship, which does not contribute to survey error, and to avoid one-to-many relationships, the initiative allowed a phone number to be registered only once. However, it was inevitable that a person with several phones (many-to-one) could send data from all of them, whether this person was registered or not.

**Fig. 3:**
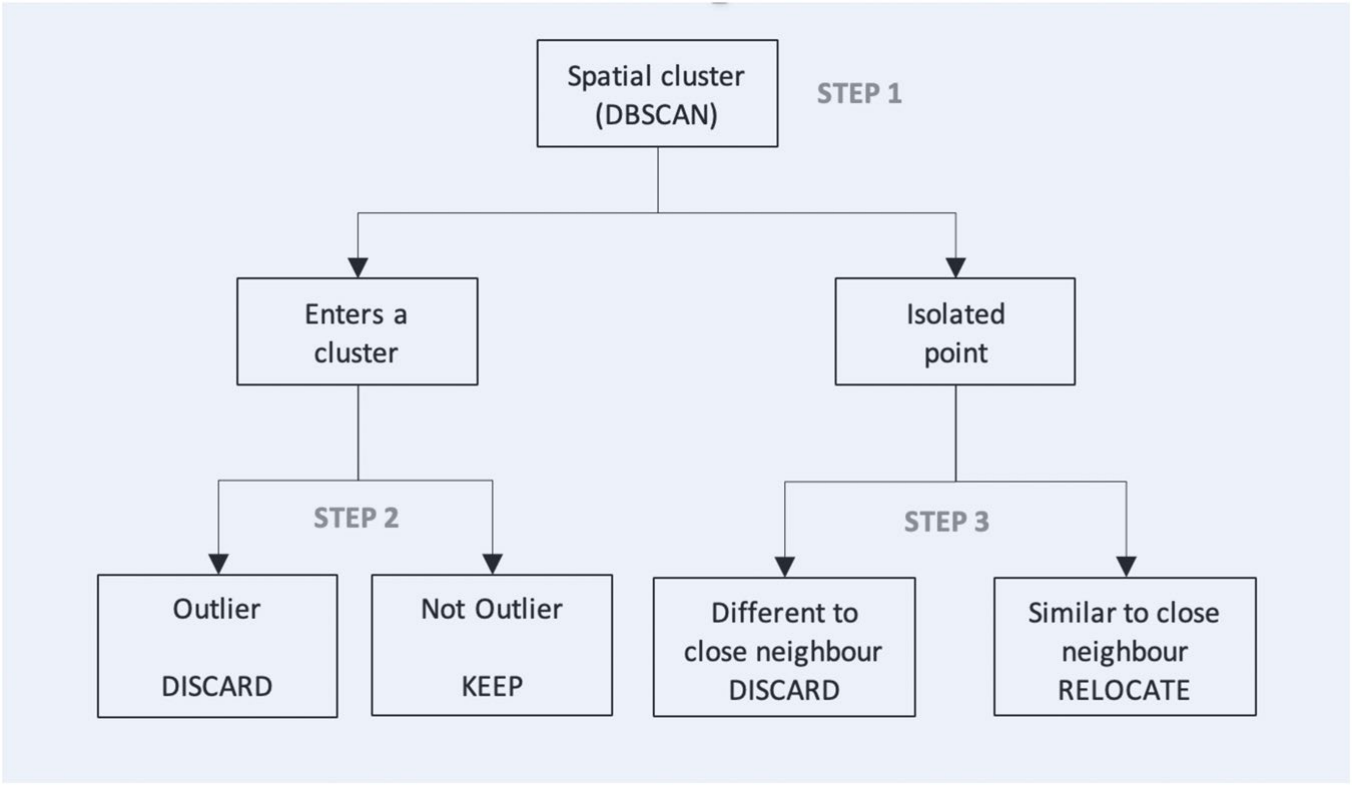
Diagrammatic representation of the three steps of outlier detection.

In order to correct non-sampling errors in data submissions, such as measurement errors or possible fraudulent activities, we ran a pre-processing routine. The pre-processing routine consisted of extracting and validating the raw data reaching the crowdsourcing platform from the mobile app in real time. This phase consists of four steps: (1) automatic data retrieval from the digital platform through the API and conversion of the JSON into structured data, (2) data transformation (e.g. standardisation of measurement units), (3) data geo-location to different levels of administrative sub-divisions and, finally, (4) outlier detection. The latter consists of three steps, shown in the diagram in Fig. 3.

Outlier detection starts by flagging outliers based purely on spatial proximity (e.g. clusters of points within a 12 km distance), and so detecting isolated points without reference to the values observed (Step 1). The method used for cluster detection is the density-based spatial clustering of applications with noise (or DBSCAN)^44^. Compared to other algorithms, the DBSCAN can group points that are close together (points with many points nearby), discarding isolated points in low-density regions. Therefore, it is possible to define different spatio-temporal markets for each food product. Notably, DBSCAN uses two parameters. The first one is the ‘eps’, the threshold used to define how close the points must be to decide whether a point is in a cluster. The second is the ‘MinPts’, the threshold for the number of points used to classify a region as dense. Based on local competition between selling points, prices are expected to be distributed over space without significant discontinuities within market boundaries. The competitive pressure from a selling point is relevant for other selling points or stores within a few kilometres and diminishes with increasing distance^45^. Moreover, as a minimum number of observations was required in each cluster, it allowed multiple contributions for the same product to be compared, leveraging the ‘wisdom of the crowd’^46^. Typically, detecting low-quality observations in crowdsourcing relies on redundancy by comparing each contribution to other contributions asked for the same task^42^. Then, averaging is a common approach for aggregating contributions for integrative solutions, where contributions are complementary, and the value relies on their integration (conversely, in selective tasks, contributions are competitive, and only one delivers the optimal solution)^47^.

In the second step, we considered the points that enter a cluster from the DBSCAN algorithm and used two statistical methods to detect price outliers. The first method consists of the classical removal of exceptional values without considering the spatial distribution of the observation. It involves detecting and removing all values that exceed k times the standard deviation from the mean. In particular, we considered k = 2. Alternatively, a more robust approach responds to the classical right-skewed price distribution, using price medians instead of the means and interquartile ranges rather than standard deviations. We applied the latter. The second method relies on the idea that it is possible to detect outliers more precisely by introducing a spatial component and comparing only nearby points of sale within the same market. When this is done, unusual data (possibly generated by non-sampling errors) can be detected by looking at price values in the vicinity of the commodity in question. The idea is to define as neighbours all the points that are closer together than an arbitrary threshold. A spatial outlier is an observation that is statistically different from the values observed in the neighbourhood and is intuitively defined as the value that exceeds *r* times the variance from the average price (1) represented by the spatial lag (2). Then, all the observations marked as outliers are removed from the dataset.

1 $${P}_{j} > lag({P}_{j})+rsd({P}_{j})\;{\rm{or}}\;{P}_{j} < lag({P}_{j})-rsd({P}_{j})$$

2 $${\rm{where}}\;lag\left({P}_{i}\right)={\sum }_{i=1}^{n}{w}_{ij}{P}_{j}\;{\rm{and}}\;{w}_{ij}=\left\{\begin{array}{c}1\;if\;i\;and\;j\;are\;neighbours\\ 0\;otherwise\end{array}\right.$$

In the third step, we consider the points that are not part of any cluster produced by the DBSCAN algorithm, classified as isolated points. If the value observed in that point is similar to the mean of a cluster, the point is associated with that cluster even if the points are distant in space, with a maximal relocation distance defined by the parameter ‘maxd’. The underlying idea is to minimise the loss of information by connecting the isolated point to a cluster instead of deleting it. If, conversely, the isolated point is very different from the mean of any other cluster, then the point is discarded.

As a result, a validated and comprehensive dataset of daily food commodity prices was produced daily, offering relevant information on commodity prices but also on characteristics of the purchase behaviour of the citizens volunteering to be part of the crowd, such as the distance travelled to market^35^ or the type of outlet. The consistent data flow from volunteers revealed nuances of commodity price data before and after harvest, which hinted that the crowdsourcing system was reliable. Price declines were observed during the autumn harvest.

From a statistical point of view, basic price aggregates may be distinguished for different types of similar product varieties, regions and distribution/marketing channels, i.e. whether it comes from a retail, wholesale or farm gate marketing channel. Different channels give rise to different price types along the food supply chain—i.e. wholesale, retail or farm gate. The term wholesale implies selling in bulk quantities (usually to other businesses), and retail stands for selling merchandise in small quantities (usually to final consumers). Commonly, retail prices are higher than wholesale prices. The farm gate price is the product price available at the farm, excluding any separately billed transport or delivery charge^48^. Accordingly, the DBSCAN procedure is run separately for the different food product varieties, regions, and price types. The wholesale, retail and farm gate classification of each price observation is made during the data transformation step of the pre-processing phase. It is based on the type of outlet (e.g. market, neighbourhood shop or supermarket) and the quantity (e.g. 100 kg, 20 kg or 1 kg) to which it refers^49^. We observe that over the period in question, 84% of prices are collected at retail markets, 13% at wholesale and only 3% at the farm gate. Concerning outlet types, about 63% of the crowdsourced prices come from open-air markets and street outlets, 28% from traditional neighbourhood shops, and only 4% and 3% from supermarkets and directly from farmers at the farm, respectively. The rest comes from specialised stores (1%) and bulk stores (1%). It very accurately reflects the situation of marketing channels for the sale of food in Nigeria, which is dominated by open-air markets, with retail chains and supermarkets accounting for a minor percentage, even for manufactured packaged food goods^50^. This purchasing behaviour is observed in both rural and urban areas. Understanding how citizens buy food from supermarkets, open-air markets, and other retail options is important for food security and urban planning^51^. Moreover, more than three-quarters of the data are reported from rural areas, reflecting the still high to intermediate levels of rurality of the focal states in North Nigeria^52,53^. In fact, it stands out that only 1% of the observations come from the focal state in the south, Lagos, which is eminently urban (41% come from Katsina, 37% from Kano and 21% from Kaduna). A follow-up survey with onboarded volunteers in Lagos revealed that people declared to be busy to participate, and the reward was not compelling enough. Also, they stalled on participating because of scepticism or perception that the initiative may be a scam, highlighting the importance of trust for crowd participation. They suggested that intensifying publicity on social media could help. Considering food products, 31% of observations are for rice (16% and 15% for local and imported rice, respectively), 23% are for beans, 20% are for maise, 15% for garri and 11% for soybeans. In terms of product quality, for those products for which the quality grade was collected, 94% of observations were from high-quality grades (i.e. 1 and 2), suggesting a preference in the crowd for higher-quality and more expensive varieties or a higher presence in the market. The data reveals that 66% of price observations were reported by volunteers merely observing the prices and not buying or selling. For more information about the FPCA data collection methodology, please refer to^49^.

### Post-sampling

As stated in the introduction, crowdsourced data is submitted voluntarily, so collecting crowdsourced data represents convenience sampling that does not obey any probabilistic design. Consequently, it is extremely risky to use it if we wish to draw reliable statistical inferences. The strategy employed in this study to tackle this problem consists of subjecting the crowdsourced data to a process of reweighting before using it in subsequent analysis and inference. In its simplest form, reweighting assigns appropriate weights to each sample unit based on inclusion probability (if known) or based on available information to obtain a distribution more similar to the population we want to investigate. When inclusion probabilities are known, we call the process *post-stratification*^28^, which is a common strategy in statistics, although its properties have received little attention^29^. In this last case, after choosing an appropriate variable whose distribution is known in the population, sample units are weighted with the ratio between the theoretic proportion (in the population) and the observed proportion in the sample. Following this basic idea^34^, suggested transforming crowdsourced datasets to resemble a predefined geographical formal sample design with reference to the space where data is collected. This particular form of post-stratification was termed *spatial post-sampling*.

In a nutshell, the s*patial* post-sampling method can be described as follows. Suppose that a set of *N* observations is collected by crowdsourcing on a set of *L* given geographical sub-areas (e.g. LGAs) into which the entire study area (i.e. the state) is partitioned. To implement the strategy, we then compare the location of the observed data with that of a set of points selected using a reference formal sample design of an equivalent sample size. While, in principle, any design can be used, it is reasonable to assume a stratified random sample with geographical stratification and probability proportional to size (pps) or one of the optimal spatial sample designs described in the literature as a reference sample design^54,55^.

In each of the L sub-areas considered, the N observations are then reweighted to resemble the formal sampling scheme by following these operational steps:

In Step 1, we count the number of observations available at a given geographical level. We will assume that in the *l-th* location (*l* = *1,…, L*), we have a total number of *n*_*l*_ crowdsourced observations, with ${n}_{l}={\sum }_{m}{n}_{m,l}$, m being the internal index of location l. The total number of observations in the whole crowdsourced exercise is ${\rm{N}}={\sum }_{l=1}^{L}{n}_{l}$. We also define *X*_*m.l*_ as the *m-th* observation of the variable of interest X in the sub-area *l*.

In Step 2, the observations in each of the L locations are averaged with a simple unweighted mean ${\bar{X}}_{l}=\frac{{\sum }_{m}{X}_{m,l}}{{n}_{l}}$.

In Step 3, we count the number of data points needed to satisfy a formal sampling procedure in each of the L locations. Using, for instance, a random stratified sample with geographical stratification and probability proportional to the population size, we can identify a sample of data points exactly equal to those observed. We define *m*_*l*_ as the number of observations which should be required by the formal design in each location, with $N={\sum }_{l=1}^{L}\,{m}_{l}$.

In Step 4 we build up a *spatial post-sampling ratio*, defined as the ratio between the number of observations required by the reference sampling plan and the number of observations actually available with crowdsourcing in each location, that is: $P{S}_{l}=\frac{{m}_{l}}{{n}_{l}}$.

Finally, in Step 5 the mean of the target variable X is calculated as a weighted average of X using the post-sampling ratio as weights. So, formally, we have:

3 $${\bar{X}}^{ps}=\frac{{\sum }_{l=1}^{L}\,P{S}_{l}\,\ast \,{X}_{l}}{{\sum }_{l=1}^{L}\,P{S}_{l}}$$

Thus, if in each location *l, PS*_*l*_ = 1, then the number of observations available in location 1 is precisely that required by the reference sampling plan, and no adjustment is needed. Conversely, if in location *l*, *PS*_*l*_ ≠ 1, then the number of observations available in location *l* is different from that required by the reference sampling plan, and the observations need to be reweighted. If no observations are available in location *l* (*n*_*l*_ = 0), then the location is not considered in the averaging process; if no observations are required in location *l* (*m*_*l*_ = 0), then the observations collected in location *l* will also not contribute to the calculation of the global mean.

In its essence, our method falls within the class of post-stratification methods, which share the idea of reweighting observations to correct for under- or over-representation and differs only in how the weights are derived. In our case, the reference to the geographical space of the collection is essential to the method.

Our framework can also be used to measure the reliability of a crowdsourcing exercise by comparing the available dataset with that required by a reference sample design.

A possible reliability measure is the following Crowdsourcing Reliability Index:

4 $$CRI=1-\frac{{\sum }_{l=1}^{L}{({m}_{l}-{n}_{l})}^{2}}{{\sum }_{l=1}^{L}{n}_{l}^{2}-2N\,\mathop{{\min }}\limits_{l}({n}_{l})+{N}^{2}}$$

with all symbols already introduced. Expression (4) is a measure of reliability that ranges between 0 and 1. In fact, in the case of low reliability, we are in the worst-case scenario when all crowdsourced data is concentrated in one single spatial sub-area where, following a formal sample design, we needed the minimum number of points. In this case ${n}_{l}=N,if\hspace{-0.15mm}l=\mathop{\min }\limits_{l}({m}_{l})$ and *n*_*l*_ = 0, otherwise and *CRI* = 0. Conversely, in the case of maximum reliability (when the crowdsourced data and the formal design perfectly coincide and we do not need any post-sampling correction), we have $({m}_{l}={n}_{l}),\forall l$ so that ${\sum }_{l=1}^{L}{\left({m}_{l}-{n}_{l}\right)}^{2}=0$ and *CRI* = 1.

The *CRI* index described above is be calculated by including all sub-areas present in the study area. If all observed data points are concentrated in one sub-area of the state, the *CRI* indicator is very low because actual data would not cover most of the region (i.e. state). Conversely, if data is collected in all sub-areas, but there are only a few observations in each area (in extreme cases only one), then *CRI* is high because data is reasonably well distributed.

## Data Records

Two types of datasets were produced and stored in https://zenodo.org/record/7261389^56^: the pre-processed dataset and the final datasets. The pre-processed dataset—step2.csv— consists of the daily raw price observations recorded between April and November 2021 by the crowd volunteers, transformed and geolocated, and from which outliers (possible non-sampling errors) have been removed, as explained in Fig. 3. The final datasets—step3_sps.csv and step3_gps_agg.csv—consist of the price observations of the pre-processed dataset, aggregated weekly and by region (i.e. state) according to the post-sampling procedure, based on a random stratified sample with pps or an optimal sample design respectively, as described in Methods.

The structure of the pre-processed dataset is related to the template developed by the FPCA team that underlaid the mobile app data submission form. This dataset includes information about the food product name, grade, packaging unit, price observed and kg price, submission date and time, position (GPS coordinates and locations) and flag for outliers or points that remain isolated (not included in a cluster). Table 1 provides an overview of the data, its description and its origin. Each row represents a price submitted for a specific food product within a data submission or data record, represented by the id_form.

**Table 1:** Pre-processed dataset field overview and description.

	Value	Description
id_form	Number	Unique identification code of a data record (automatic recording)
id	Number	Unique identification of a line (price) within a data record (automatic recording)
deviceid	Number	Device unique identification number (automatic recording)
volunteer_id	Number	Volunteer unique identification number assigned by the FPCA team (manual recording)
product	Text	Product name selected from a predefined list (manual recording)
type	Text	Product grade selected from a predefined list (manual recording)
price_type	Text	Retail, wholesale or farmgate based on market type and packaging unit (pre-processing routine)
market_type_cat	Text	Market type selected from a predefined list (manual recording) from the International Comparison Program (ICP) (https://openknowledge.worldbank.org/handle/10986/22520)
lat	Number	lat (automatic recording); unit: Decimal degrees
lon	Number	long (automatic recording); unit: Decimal degrees
alt	Number	alt (automatic recording); unit: Meters
pre	Number	pre (automatic recording); unit: Decimal degrees
level0	Text	Country (pre-processing routine) from UN Office for the Coordination of Humanitarian Affairs (https://data.humdata.org/dataset/cod-ab-nga)
level0code	Text	Country code (pre-processing routine) from UN Office for the Coordination of Humanitarian Affairs (https://data.humdata.org/dataset/cod-ab-nga)
level1	Text	State (pre-processing routine) from UN Office for the Coordination of Humanitarian Affairs (https://data.humdata.org/dataset/cod-ab-nga)
level1code	Alphanumeric	State code (pre-processing routine) from UN Office for the Coordination of Humanitarian Affairs (https://data.humdata.org/dataset/cod-ab-nga)
level2	Text	LGA (pre-processing routine) from UN Office for the Coordination of Humanitarian Affairs (https://data.humdata.org/dataset/cod-ab-nga)
level2code	Alphanumeric	LGA code (pre-processing routine) from UN Office for the Coordination of Humanitarian Affairs (https://data.humdata.org/dataset/cod-ab-nga)
time_start	Date/time	Start time of data submission (automatic recording)
submission_time	Date/time	Submission time (automatic recording)
packaging	Text	Packaging unit selected from a list (manual recording)
conversion	Number	Conversion factor (pre-processing routine)
price_observed	Number	Price observed in Naira (manual recording)
price_kg	Number	Price converted to kg price in Naira (pre-processing routine)
buying_purpose	Text	Selected from a predefined list (e.g. for consumption, for selling, only price observer, etc.) (manual recording)
market_distance	Number	Distance in km from home to market (manual recording)
area	Number	Surface area in metres of the LGAs
population	Number	Population data at LGA level
density	Number	Calculation (pre-processing routine)
level3urban_new	FALSE/TRUE	Urban-rural area identification (pre-processing routine)
outlier	FALSE/TRUE	Outlier points (pre-processing routine)
cluster	Number	Cluster number where zero is assigned to the isolated points (pre-processing routine)
relocation	FALSE/TRUE	Relocated points (pre-processing routine)

Table 2 presents the list of food products, the number of prices reported for each product and their share in the total valid observations.

**Table 2:** List of food products and the corresponding amount of observations.

Food product	n observations	% observations
local_rice	37,013	16.07
indian_rice	12,608	5.47
thailand_rice	21,694	9.42
maize_white	25,966	11.27
maize_yellow	21,497	9.33
red_beans	18,707	8.12
white_beans	33,936	14.73
white_garri	21,199	9.2
yellow_garri	13,041	5.66
soybean	24,674	10.71
Total	230,335	100.00

Tables 3, 4 show the packaging units and market types selectable from the tool, respectively, and the number of observations for each packaging unit.

**Table 3:** List of packaging units and the corresponding amount of observations.

Packaging unit	n observations	% observations
Mudu/Kwano	188588	81.87
Kongo	255	0.11
1 kg	438	0.19
5 kg	317	0.14
10 kg	84	0.04
25 kg	364	0.16
50 kg	11970	5.2
100 kg	28319	12.29
Total	230335	100

**Table 4:** List of market types and the corresponding amount of observations.

Market type	n observations	% observations
Bulk and discount stores	2652	1.15
Directly from farmer	7074	3.07
Supermarket	8552	3.71
Open-air markets	137090	59.92
Neighbourhood shop	63730	27.67
Specialised stores	3172	1.38
Street outlets	8065	3.5
Total	230335	100.00

The structure of the final datasets (Table 5) is based on Arbia *et al*. (2018). These datasets result from the post-sampling procedure and include information about the region, the submission week and year, the product and price type, the weekly simple average and the post-sampled average and the crowdsourcing reliability indicator. Each row represents the weekly price of a specific food product from a marketing channel type (i.e. retail, wholesale, farm gate).

**Table 5:** Final dataset field overview and description.

Field	Value	Description
level1	Text	State
submission_week	Number	Week number
submission_month	Number	Month number
submission_year	Number	Year
week_start	Date	Week start date
product	Text	Product name
price_type	Text	Price type
price.mean	Number	simple price average
price.ps	Number	post-sampled (weighted) average
CRI	Number [0, 1]	Crowdsourcing reliability indicator

## Technical Validation

The main objective of this work is to test the possibility of producing and using reliable real-time data from crowdsourcing (i.e. mobile app-contributed data) on food prices. Crowdsourcing can provide more timely information at a more granular level. For this purpose, in our case study, we employ the procedure described in Methods to clean and post-sample the crowdsourced food price data collected in Nigeria.

To do this, the raw data of the crowdsourcing platform go through the quality procedure implemented in a series of codes and algorithms fully developed in the R software^57^ in two phases. First, the pre-processing phase goes from extracting the data through the platform API and transforming them by cleaning the outliers due to possible data entry errors by voluntary participants (non-sampling error). The output of this phase is the pre-processed dataset. Secondly, the post-sampling phase corrects for the potential sampling error inherent to crowdsourcing data collection. Individual observations are weighted according to the two proposed strategies to make the dataset resemble a formal sample design (i.e. a random stratified sample design with probability proportional to population and an optimal spatial sampling design) to produce reliable and accurate estimates. Besides, the CRI (Crowdsourcing Reliability Index) indicator provides a normalised measure of the coverage of the crowdsourcing data points compared with the formal sample design. The output of this phase are the final datasets. As a result, the price data is updated twice a day on an interactive web dashboard^37^. The R codes for all these operations are available at https://github.com/vincnardelli/fpca.

In the pre-processing phase, we adopted a procedure with customisable parameters to build dynamically spatial clusters or “spatial-temporal markets” (using, e. g., DBSCAN; Ester *et al*. 1996) and then identify within-cluster price outliers. As a result, outliers and isolated points (those that could not be joined to any cluster) are removed. Setting the parameters eps = 0.0019 (~12 km), MinPts = 5, maxd = 0.0078 (~50 km), over the eight months of weekly analysis (April to November 2021), we found 9,651 (or 4%) outliers and 65,628 (or 29%) isolated points. However, there are differences between commodities and states. Katsina has the highest percentage of valid observations (75%), with only 20% isolated points and 4% outliers. Kano is in second place with 69% valid observations, 27% isolated points and 4% outliers. Then there is Kaduna, with 51% accurate observations, 45% isolated points and 4% outliers. Finally, with considerably fewer observations in Lagos State, there are 48% valid observations, 50% of isolated points and 2% outliers. Over time, the percentage of outliers decreases, while the share of isolated points grows as the number of submitted prices consistently diminishes. It may reflect the effect on the quality of the monetary reward (only paid for valid submissions) and that voluntary participants learn and reduce input errors over time to maximise the reward’s probability. Moreover, on the other hand, the volunteers’ motivation seems to diminish over time, indicating the need to communicate with the crowd and implement strategies beyond financial incentives to keep volunteers engaged^36^.

Next, we apply the suggested methods of estimation. We ran the post-sampling procedure and compared the performances of the different strategies^34^. As a result, we obtained three price estimates (FPCA estimates). The first assumes the crowdsourcing sample as a simple random sample and estimates the mean of the variable *X* (commodity price) for each state (first level of geographic aggregation) with the unweighted Horvitz-Thompson estimator. Let us call this estimator ${\widehat{X}}_{FPCA}^{HT}$ (FPCA-HT). Since we know that crowdsourcing is a non-probabilistic sampling process, this choice can lead to biased and highly inefficient estimates for price, because it neglects the different densities with which price data are distributed over space. The second strategy uses the Horvitz-Thompson estimator ${\widehat{X}}_{FPCA}^{PSHT}$ (FPCA-PSHT) with the individual crowdsourcing observations, but now weighted using the post-sampling (PS) ratios obtained by comparing the actual data with a random stratified design, with probability proportional to the population of the LGAs (second level of geographic aggregation). Finally, the third strategy again uses the Horvitz-Thompson estimator ${\widehat{X}}_{FPCA}^{SPSHT}$ (FPCA-SPSHT) and post-sampling correction, but using the spatial post-sampling ratios obtained by comparing the actual data with an LPM2 design^55^. No auxiliary variable is required for the third strategy except for the geo-coordinates of each data point, which are recorded automatically at data submission by the mobile app. Notice that the acronym PS was used in a generic sense in Methods section to denote the post-sampling strategy, while here we distinguish between a PS strategy (when we use a random stratified sample as a benchmark) and an SPS strategy (when we use a spatial sampling strategy as reference).

We assume that both post-sampled estimates $\left({\widehat{X}}_{FPCA}^{PSHT}\;{\rm{and}}\;{\widehat{X}}_{FPCA}^{SPSHT}\right)$ are more accurate and efficient estimators than the simple average $\left({\widehat{X}}_{FPCA}^{HT}\right)$. Accuracy refers to the difference between the estimate and the ‘true’ value of the parameter of interest.

Trust in crowdsourcing data is a fundamental issue for future data collection and analysis applications. Indeed, the increasing quantity of data available through technologies and mobile apps represents the main advantage of crowdsourcing and participatory approaches such as citizen science. However, this makes it necessary to both establish trusted algorithms which can harvest the information and produce relevant data in a timely fashion, as well as to accurately describe the uncertainty associated with such algorithms. Crowdsourcing and technology provide easy access to a large amount of data from across multiple geographies in real-time. However, a drawback is that it relies on “convenience sampling”, an example of non-probability sampling, which typically leads to possible problems with representation. The validity of the crowdsourcing approach depends on several factors: (i) the target population’s (the crowd’s) availability of and access to technology, (ii) the involvement and motivation of the crowd, (iii) the number of valid data points, and their spatial dispersion, which can compromise the quality and representation of the information; and (iv) the use of an adequate procedure to deal with the over or under-representation of the sample units^34^.

This paper focuses on the last two points, proposing a methodology to trust the crowdsourced data collection by applying a validation through pre-processing algorithms to the data and a post-sampling strategy approach to deal with the bias and inefficiencies associated with non-probabilistic samples (Section on Methods). On both point (iii) and (iv) above, spatial auxiliary information of the data (i.e. the coordinates of the price data points) was used to improve the ‘trust’ in the data and in the final estimate.

The ‘trusted’ methodology proposed was validated and tested through an application in Nigeria concerning mobile-app-based crowdsourcing to collect daily food market prices: nine food product varieties in the states of Kano, Katsina, Kaduna and Lagos. The three price estimates resulting from the crowd data collection were obtained by respectively applying a Horvitz-Thompson estimator, the same but improved by weighting with population data and by weighting considering the spatial location of the data.

The proposed methodology could be extended by including other alternative data sources on prices, including geographic information (e.g. web scraped data). Moreover, it would be worthwhile to explore how this methodology could be integrated with data obtained through classical statistic approaches, for example, to produce accurate short-term forecasts.

From a practical perspective, we are confident that the proposed dataset and methodology could be extremely useful for institutions and organisations aiming to complement price data collection systems with real-time and highly granular data approaches.

### Depositing your data to an appropriate repository

The dataset(s) described therein have been deposited in a public repository Joint Research Centre Data Catalogue and available at the following link: https://data.jrc.ec.europa.eu/dataset/f3bc86b0-be5f-4441-8370-c2ccb739029e.

## Data Availability

Code for data pre-processing and post-sampling is developed in R and available at https://github.com/vincnardelli/fpca and https://zenodo.org/record/7261389.
